# Effects of Isoxazolo-Pyridinone 7e, a Potent Activator of the Nurr1 Signaling Pathway, on Experimental Autoimmune Encephalomyelitis in Mice

**DOI:** 10.1371/journal.pone.0108791

**Published:** 2014-09-29

**Authors:** Francesca Montarolo, Chiara Raffaele, Simona Perga, Serena Martire, Annamaria Finardi, Roberto Furlan, Samuel Hintermann, Antonio Bertolotto

**Affiliations:** 1 Neurobiology Unit, Neurologia 2 – CRESM (Regional Referring Center of Multiple Sclerosis), Neuroscience Institute Cavalieri Ottolenghi (NICO), University of Turin and AOU San Luigi, Orbassano, Torino, Italy; 2 Division of Neuroscience, Experimental Neurology Institute (INSPE), San Raffaele Scientific Institute, Milan, Italy; 3 Global Discovery Chemistry, Novartis Institutes for BioMedical Research, Basel, Switzerland; Friedrich-Alexander University Erlangen, Germany

## Abstract

Multiple sclerosis (MS) is an autoimmune chronic disease of the central nervous system (CNS) characterized by immune-mediated inflammation, demyelination and subsequent axonal damage. Gene expression profiling showed that Nurr1, an orphan nuclear receptor, is down-regulated in peripheral blood mononuclear cells of MS patients. Nurr1 exerts an anti-inflammatory role repressing the activity of the pro-inflammatory transcription factor NF-kB. Here, we report that the preventive treatment with isoxazolo-pyridinone 7e, an activator of Nurr1 signaling pathway, reduces the incidence and the severity of a MS murine model, i.e. experimental autoimmune encephalomyelitis (EAE). The compound is able to attenuate inflammation and neurodegeneration in spinal cords of EAE mice by an NF-kB pathway-dependent process.

## Introduction

The Nuclear receptor related 1 protein (Nurr1) also known as NR4A2 belongs to the NR4A subfamily consisting of three members: NR4A1 (Nur77), NR4A2 (Nurr1), and NR4A3 (Nor-1). Although Nurr1 appertains to the steroid nuclear hormone receptor class, it is considered an orphan receptor because its activity is not regulated by ligands and its structure is locked in a constitutively active form [1].

Nurr1 is essential for the development of midbrain dopamine neurons [2]–[5] with complete agenesis of midbrain dopamine cells apparent at birth in Nurr1 knockout mice. Three point mutations in Nurr1 gene were found in association with a familiar form of Parkinson Disease (PD) [6] and down-regulated gene expression levels were found in brains of aged individuals [7] and in peripheral blood mononuclear cells (PBMCs) [8] of PD patients with progressive loss of dopaminergic neurons.

Furthermore, Nurr1 plays an anti-inflammatory role by inhibiting the expression of inflammatory genes in microglia and astrocytes [9]. In particular, Nurr1 cooperates with a complex of nuclear proteins in the CoREST-dependent trans-repression pathway to repress the activity of the pro-inflammatory transcription factor NF-kB [9]. In fact, knocking down Nurr1 with small hairpin RNA in mice, glial cells exposed to lipopolysaccharide (LPS) become more active, producing higher levels of inflammatory cytokine-encoding mRNAs and neurotoxic effector proteins such as inducible nitric oxide synthase 2 [9].

Increasing evidence suggests also a role of Nurr1 in inflammatory responses in autoimmune pathologies such as arthritis [10] and psoriasis [11]. The role of Nurr1 in Multiple Sclerosis (MS) is controversial [12], [13]. MS is an heterogeneous autoimmune chronic disease of central nervous system (CNS) characterized by immune-mediated inflammation, demyelination and subsequent axonal damage [14], [15]. Previous works report either down-regulation or up-regulation of Nurr1 gene expression in PBMCs in Caucasian [13] or Japanese [12] MS patients, respectively.

Regarding the role of Nurr1 in MS, we previously characterized a gene signature of PBMCs obtained from MS patients in which Nurr1 resulted significantly down-regulated with respect to healthy controls [16]. Nurr1 gene deregulation was confirmed by our following studies [17], in which we also observed that the gene expression level correlates with the aggressiveness of the pathology and clinical parameters such as the relapse rate and the Expanded Disability Status Scale (EDSS) progression [17]. In particular, we reported that the Nurr1 expression level went back to normal in pregnant MS patients paralleling the clinical remission. Recently, our laboratory also reported a fundamental role of CD4+ T cells and monocytes in Nurr1 gene expression down-regulation in MS patients [18].

It is now common knowledge that both CD4+ Th1 and Th17 cells mediate autoimmune responses in human MS [19]–[21] as well as in its animal model, i.e. experimental autoimmune encephalomyelitis (EAE) [22]–[24]. The mechanisms leading to this kind of disorder are not yet understood. Last year, Raveney and coworkers reported that Nurr1 exerts a key role in Th17 differentiation and that Nurr1 knock-down *in vivo* by injection of Nurr1 small interfering RNA reverses autoimmune responses and ameliorates clinical symptoms in EAE [25].

In this study we show the effect of a highly potent brain-penetrable activator of the Nurr1 signaling pathway in a murine model of MS (MOG_35–55_ induced EAE). To date, only three activators of the Nurr1 signaling pathway have been reported: 6-mercapto-purine [26], 1, 1-bis(3-indolyl)-1-(p-chlorophenyl) methane (DIM-C-pPhCl) (6-MP) [27] and isoxazolo-pyridinone 7e (IP7e) (6-(4-((2-methoxyethoxy)methyl)phenyl)-5-methyl-3-phenylisoxazolo(4,5-c)pyridin-4(*5H*)-one) [28]. We decided to use IP7e since the others have many additional cellular activities, including anti-proliferative and cytotoxic effects due to inhibition of purine biosyntesis [26]. IP7e also has excellent oral bioavailability in mice, rapid and extensive brain uptake [28] and high specificity for Nurr1 and not for the NR4A subfamily [28].

This is the first report of preventive and therapeutic effects of IP7e in EAE. We demonstrate that the preventive administration of IP7e delays the onset and reduces the incidence and severity of EAE, and decreases neuroinflammatory and histopathological alterations in the spinal cord of treated EAE mice. On the contrary, the course of EAE is not influenced by the therapeutic administration. Finally, the preventive administration of IP7e induces down-regulation of NF-kB downstream genes in the spinal cord suggesting an inhibiting action on the NF-kB signaling due to activation of the Nurr1 pathway when the substance is administered before EAE onset.

## Materials and Methods

### 2.1 Animals

The animal experimental procedures were approved by the Bioethic Committee of the University of Turin (prot. September 26, 2013) and were communicated and authorized by the Ministry of Health. All experiments were carried out according to the European Union Directives 86/609/EEC and 6106/10/EU.

Female C57BL/6J mice for all the experiments were purchased from Charles River (Calco, Italy). Mice were housed in pathogen-free conditions with a light/dark cycle of 12 h and free access to food and water.

### 2.2 EAE induction and clinical evaluation

In order to induce EAE 6–8 week-old-female mice were immunized by subcutaneous injection under the rostral part of the flanks and at the base of the tail with 300 µl of 200 µg/mouse of myelin oligodendrocyte glycoprotein (MOG_35–55_; Espikem, Florence, Italy) in incomplete Freund's adjuvant (IFA; Sigma-Aldrich, Milan, Italy) containing 8 mg/ml *Mycobacterium tuberculosis* (strain H37Ra; Difco Laboratories Inc., Franklin Lakes, NJ, USA), followed by two intravenous injections of 500 ng of *Pertussis* toxin (Duotech, Milan, Italy) on the immunization day and 48 h later. Body weight and clinical score (0 = healthy; 1 = limp tail; 2 = ataxia and/or paresis of hind limbs; 3 = paralysis of hind limbs and/or paresis of forelimbs; 4 = tetraplegia; 5 = moribund or dead) were recorded daily by an investigator blind to group identity. Median clinical score and interquartile range (IR) were calculated for each group per day to analyze the time course of EAE. Cumulative and maximum score and mean body weight were calculated. The percentage of disease-free mice was calculated evaluating the day post immunization (d.p.i.) when the first clinical manifestations appeared (score>0).

### 2.3 IP7e administration (activator of the Nurr1 signaling pathway)

The activator of the Nurr1 signaling pathway (isoxazolo-pyridinone 7e; IP7e, Novartis, Basel, Switzerland) [28] was dissolved in Tween 80 in a 10× stock solution. To obtain the final concentration (1×; 10 mg/kg) IP7e was dissolved in saline solution (0.9% NaCl).

Two kinds of treatment by gavage were performed twice a day: preventive administration (before the disease onset) from 7 to 23 d.p.i. and therapeutic (after the disease onset) from 21 to 36 d.p.i. Control animals received the Tween 80 dissolved saline solution (0.9% NaCl, vehicle) twice a day.

### 2.4 Histological evaluation

At the end of treatments, mice were deeply anesthetized and transcardially perfused with saline EDTA followed by cold 4% paraformaldehyde in 0.1 M phosphate buffer (pH 7.4). Spinal cords were removed and after 24 hours of post-fixation in 4% paraformaldehyde were washed in phosphate buffered saline (PBS) and embedded in paraffin. To quantify neurological damage in EAE mice, paraffin-embedded 10 µm sections were stained with Bielshowsky, Luxol fast Blue, and Hematoxylin and Eosin to detect the presence of axonal loss, demyelination and perivascular inflammatory infiltrates, respectively. Macrophages were stained with biotin-labeled BS-I isolectin B4 (Sigma, St Louis, MO). T cells were stained using a rat anti-CD3 (pan-T cell marker, Serotec Ltd, Oxford, UK) revealed with a biotin-labeled secondary anti-rat antibody (Amersham, UK). Neuropathological findings were quantified in an average of 12 complete cross-sections of spinal cord per mouse representative of whole spinal cord levels. Demyelination and axonal loss were expressed as percentage of damaged area per mm^2^, the number of perivascular inflammatory infiltrates was calculated and expressed as the numbers of perivascular inflammatory infiltrates per mm^2^, T cells and macrophages were counted and expressed as number of cells per mm^2^.

### 2.5 Biomolecular analysis

At the end of treatments, mice were deeply anesthetized and transcardially perfused with saline EDTA and spinal cords and brains were removed. All samples were rapidly frozen and total RNA was isolated by extraction with the TRIzol Reagent (Invitrogen Life Technologies, Carlsbad, CA, USA) according to the manufacturer's instructions. Total RNA was reverse-transcribed to complementary DNA (cDNA) using the Ready-To-Go You-Prime First-Strand Beads (Amersham, Airlington Heights, IL, USA) and Random Hexamer (New England Biolabs, Ipswich, MA, USA) according to the manufacturer's instructions. cDNA was used as a template for a real-time PCR analysis. Quantitative Real-time PCR was carried out using the ABI Prism 7000 Sequence Detection System (Applied Biosystems, Life Technology, Paisley, UK). The expression of 84 target genes of the NF-kB pathway were analyzed using the RT^2^ Profiler PCR Array mouse NF-kB Signaling Target (PAMM-225Z) according to the manufacturer's protocol.

### 2.6 Statistics

Data are presented as median value ± interquartile range (IR), median alone or mean ± standard error of mean (SEM). Unless otherwise indicated, n = number of animals. Statistical significance was assessed by Student's *t*-test (Mann–Whitney post hoc test) to evaluate the percentage of disease free mice, cumulative and maximum score, and the presence of inflammatory/neurophatological alterations. Two Way ANOVA, was performed to evaluate clinical score and body weight changes during the period of treatment. All the graphs and statistical tests were performed and designed using Graph Pad Prism software (San Diego California, USA). *P* values less than 0.05 were accepted as significant.

## Results

### 3.1 Preventive IP7e treatment ameliorates EAE

**Figure 1:**
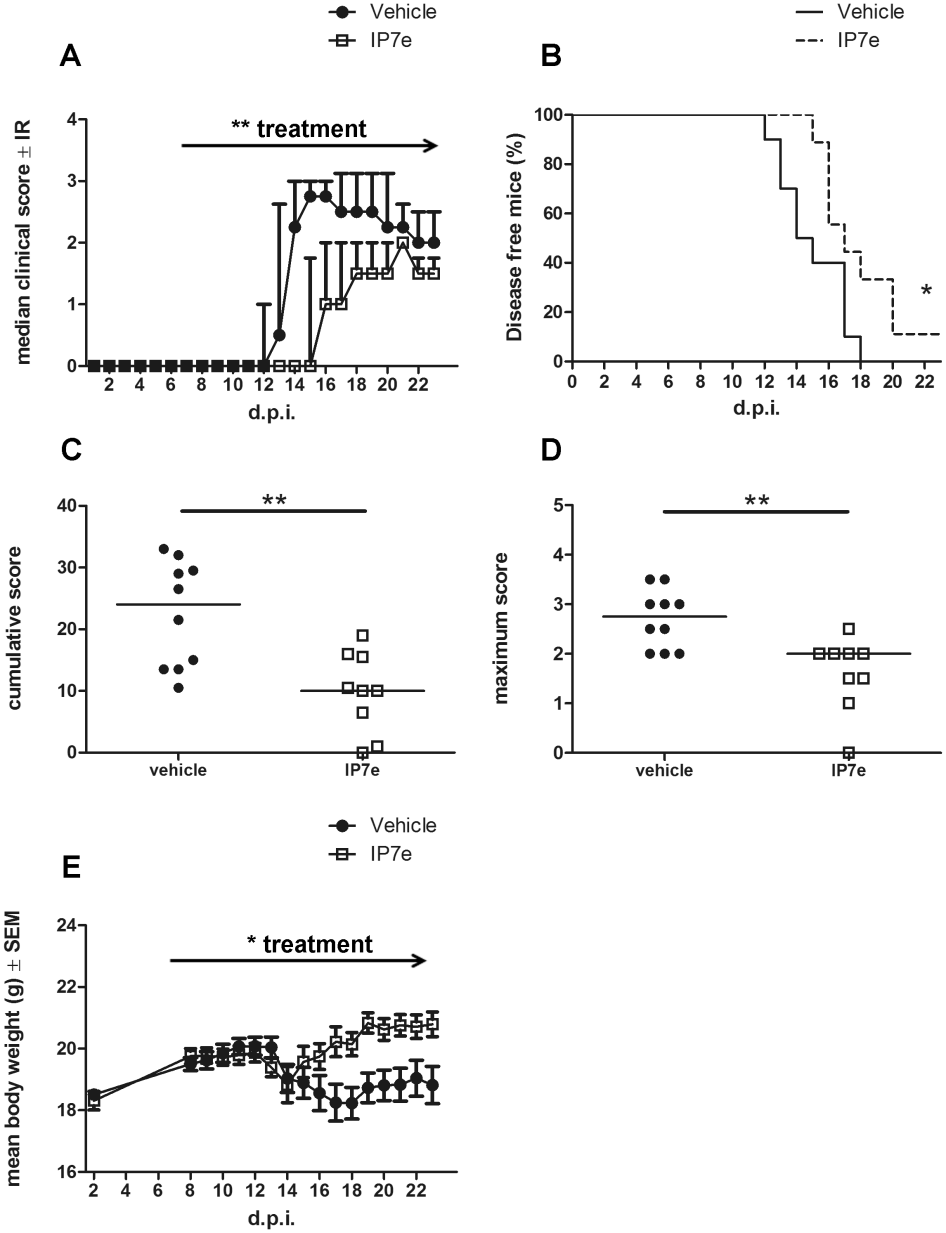
Preventive administration of IP7e reduces the severity and the incidence of EAE. The clinical course of EAE in IP7e (n = 9) and vehicle (n = 10) -treated EAE mice is compared. The analysis shows a decreased clinical score, i.e. median course (A), cumulative (C, bar represents median value) and maximum (D, bar represents median value) clinical score and a reduced weight loss (E) after the preventive administration of IP7e in EAE mice. IP7e leads also to an increase in percentage of disease-free mice (B). Arrows indicate the period of treatment (from 7^th^ to 23^th^ d.p.i.). Data are representative of 2 independent experiments. (Fig. A; Two Way ANOVA, treatment **P<0.01, Fig. E; Two Way ANOVA, treatment *P<0.05, Figure B, C, D; Student's *t* test, *: *P*<0.05; **: *P*<0.01). (d.p.i.; day post immunization, IP7e; isoxazolo-pyridinone 7e, IR; interquartile range, SEM; standard error of mean).

To evaluate the effect of IP7e on the onset of MOG_35–55_ -induced EAE we studied the consequences of the preventive administration of the compound on the clinical course. In the preventive treatment, immunized mice received the compound via gavage beginning from the 7^th^ d.p.i., when phenotypic EAE signs are not yet evident but the immunization process has already occurred. The analysis revealed that the IP7e preventive treatment was able to delay the disease progression (Fig. 1A; Two Way ANOVA, treatment ** *P*<0.01), as well as improve the percent of disease-free mice (Fig. 1B; Student's *t*-test, * *P*<0.05) with statistically significant reduction of cumulative (Fig. 1C; Student's *t*-test, ** *P*<0.01) and maximum (Fig. 1D; Student's *t*-test, ** *P*<0.01) disease scores compared to the vehicle-treated controls. Furthermore, the body weight was restored only in IP7e-treated mice, while vehicle-treated mice showed a massive weight loss (Fig. 1E; Two Way ANOVA, treatment * *P*<0.05).

**Figure 2:**
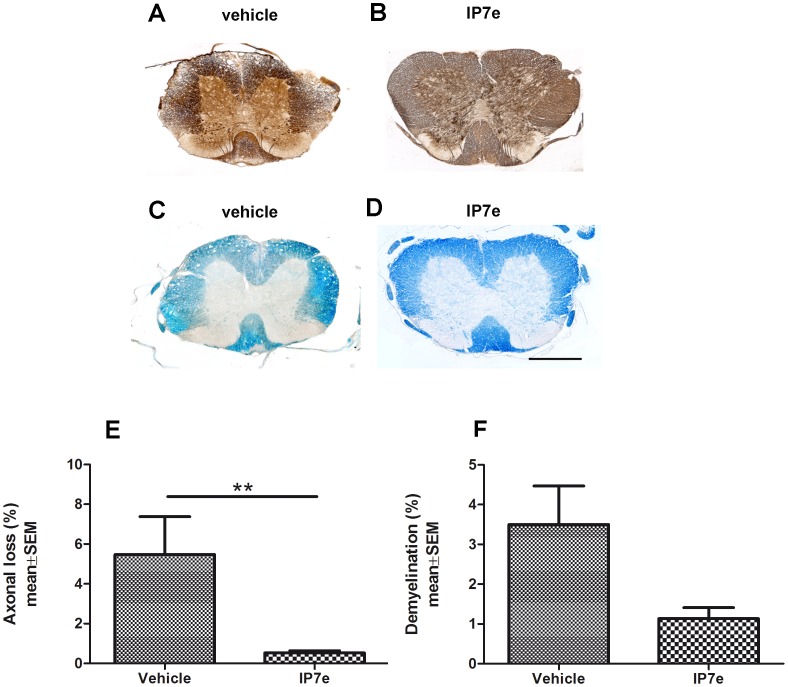
Preventive administration of IP7e reduces axonal damaged area in the spinal cord of mice with EAE. Coronal sections of spinal cord of IP7e (B, D) and vehicle (A, C) –treated EAE mice. Axonal damage and demyelination are measured using respectively the Bielshowsky (A, B) and the Luxol fast Blue (C, D) staining in the spinal cord of IP7e (n = 5) and vehicle (n = 5) -treated EAE mice. Calibration bar, 250 µm. Quantification shows that the extent of neuronal damage (E) after IP7e treatment is significantly decreased, while the extent of demyelination (F) shows a tendency to decrease after IP7e treatment compared to vehicle- treated mice. (Student's *t* test, **: *P*<0.01). Data are expressed as mean ± SEM. (IP7e; isoxazolo-pyridinone 7e, SEM; standard error of mean).

Mice receiving preventive treatment and control groups were sacrificed in the 23^th^ d.p.i., and the spinal cords were stained with Bielshowsky (Fig. 2A, B) and Luxol fast Blue (Fig. 2C, D) for the assessment of axonal damage and demyelination, respectively. Quantification of Bielshowsky staining revealed a decrease in axonal loss after treatment (Fig. 2E; Student's *t*-test, ** *P*<0.01), while demyelination was not significantly reduced, even if it showed a trend to decrease in IP7e-treated EAE mice (Fig. 2F; Student's *t*-test, *P* = 0.09).

**Figure 3:**
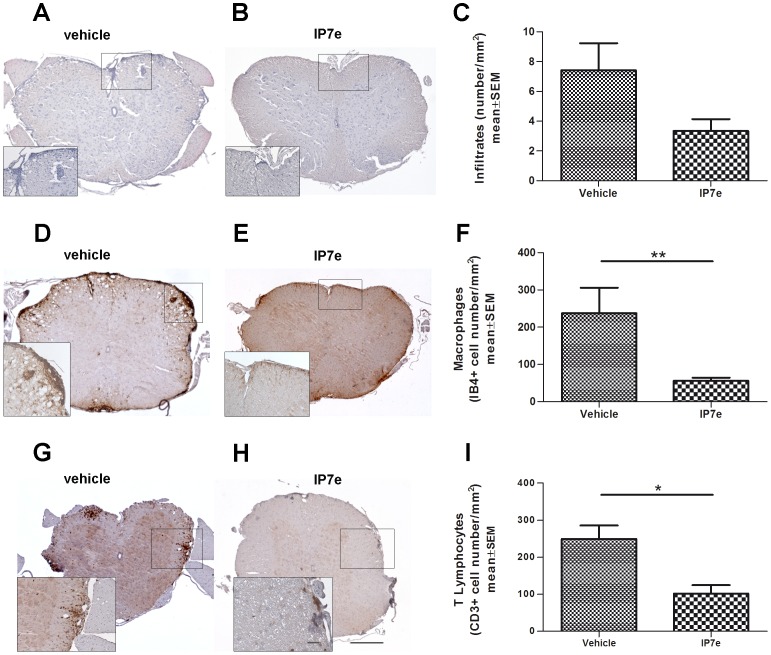
Preventive administration of IP7e reduces the number of macrophages and T lymphocytes infiltrated in the spinal cord of mice with EAE. Representative images of coronal sections of the spinal cord of IP7e (B, E, H) and vehicle (A, D, G) –treated EAE mice. The presence of perivascular inflammatory infiltrates (A, B), macrophages (D, E) and T lymphocytes (G, H) are evaluated using respectively Hematoxylin and Eosin staining and immunohistochemistry (IB4+ and CD3+ cells for macrophages and T lymphocytes, respectively) in the spinal cord of IP7e (n = 5) and vehicle (n = 5) -treated EAE mice. Calibration bars, 25 µm and 250 µm. Quantification shows that the number per section of perivascular inflammatory infiltrates shows a tendency to decrease after IP7etreatment (C). Furthermore, the number per section of macrophages (F) and T lymphocytes (I) after IP7e treatment results significantly decreased compared to vehicle-treated mice. (Student's t test, *: *P*<0.05; **: *P*<0.01). Data are expressed as mean ± SEM. (IP7e; isoxazolo-pyridinone 7e, SEM; standard error of mean).

As shown in Figure 3, IP7e treatment did not significantly decrease perivascular inflammatory infiltrates, but caused a trend to reduction (Fig. 3A–C, Student's *t*-test, *P* = 0.09). Therefore, we decided to analyze a possible alteration in a specific cellular population of infiltrating cells. Immunohistochemistry for the assessment of the cellular composition of the perivascular infiltrates revealed that there were less macrophages (Fig. 3D–F; Student's *t*-test, ** *P*<0.01) and T lymphocytes (Fig. 3G–I; Student's *t*-test, * *P*<0.05) in the IP7e than in vehicle-treated animals.

These results suggest that preventive administration of IP7e, starting before the EAE onset, reduces the severity of the disease with a general improvement of clinical signs and neuropathological features.

### 3.2 Therapeutic IP7e treatment does not improve EAE

**Figure 4:**
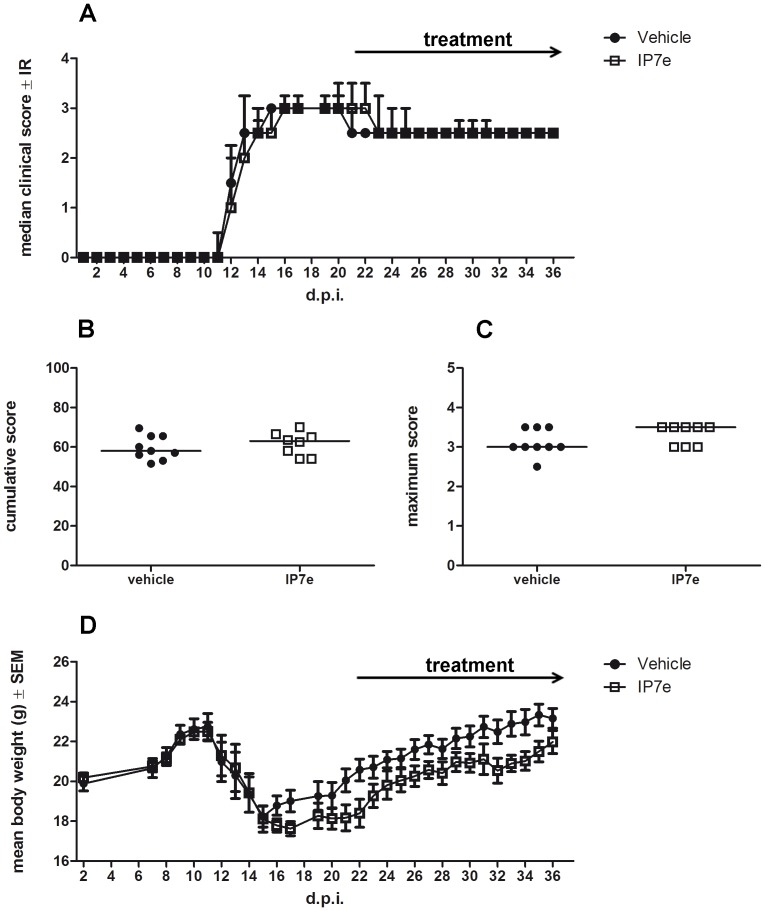
Therapeutic administration of IP7e does not influence the severity of EAE. The clinical course of EAE in IP7e (n = 8) and vehicle (n = 9) –treated EAE mice is compared. The analysis does not show any difference of disease severity i.e. median course (A), cumulative (B, bar represents median value) and maximum (C, bar represents median value) clinical score and weight loss (D) after the therapeutic administration of IP7e in EAE mice. Arrows indicate the period of treatment (from 21^th^ to 36^th^ d.p.i.). Data are representative of 2 independent experiments. (Two Way ANOVA, Student's *t* test). (d.p.i.; day post immunization, IP7e; isoxazolo-pyridinone 7e, IR; interquantile range, SEM; standard error of mean).

In order to test the possible therapeutic role of the IP7e on MOG_35–55_-induced EAE, we administered the compound after the first EAE clinical signs. Starting from the 21^th^ d.p.i., we treated EAE mice via gavage with IP7e or vehicle alone. The analysis revealed that IP7e had no effect on the clinical course (Fig. 4A; Two Way ANOVA, treatment, *P*>0.05) even considering cumulative and maximum score (Fig. 4B–C; Student's *t*-test, *P*>0.05). The body weight was not altered either (Fig. 4D; Two Way ANOVA, treatment, *P*>0.05).

**Figure 5:**
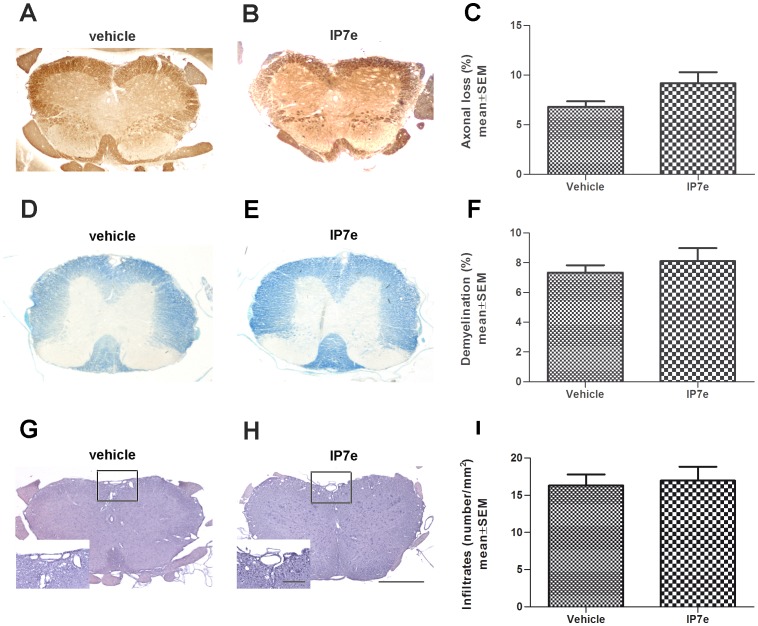
Therapeutic administration of IP7e does not influence EAE axonal loss, demyelination and, perivascular inflammatory infitration. Representative images of coronal sections of the spinal cord of IP7e (B, E, H) and vehicle (A, D, G) –treated EAE mice. Axonal damage, demyelination, and perivascular inflammatory infiltrates are measured using respectively Bielshowsky (A, B), Luxol fast Blue (D, E) and Hematoxylin and Eosin (G, H) staining in the spinal cord of IP7e (n = 4) and vehicle (n = 5) -treated EAE mice. Calibration bars, 25 µm and 250 µm. Quantification shows no differences in axonal loss (C), demyelination (F) and perivascular infiltrates (I) after the therapeutic administration of IP7e in EAE mice (Student's *t* test). Data are expressed as mean ± SEM. (IP7e; isoxazolo-pyridinone 7e, SEM; standard error of mean).

To understand whether the therapeutic administration could induce inflammatory or other neurophatological alterations in the spinal cord we sacrificed the animals on the 36^th^ d.p.i. and stained sections with Bielshowsky (Fig. 5A, B), Luxol fast Blue (Fig. 5D, E), Hematoxylin and Eosin (Fig. 5G, H). Quantification of axonal loss, demyelination and total perivascular inflammatory infiltrates revealed no differences in any of the parameters analyzed (Fig. 5C, F, I; Student's *t*-test, *P*>0.05), suggesting a lack of involvement of Nurr1 signaling activation once EAE has occurred.

### 3.3 Preventive IP7e treatment induces a down-regulation of NF-kB signaling target genes in the spinal cord of EAE mice

In order to better elucidate the role of preventive and therapeutic administration of IP7e on EAE, we performed a molecular study in Real Time PCR on the expression of NF-kB downstream target genes since the activation of Nurr1 is able to induce an inhibition on NF-kB pathway [9]. In particular we investigated the expression profile of 84 key genes responsive to NF-kB signal transduction in spinal cord and brain of IP7e and vehicle-treated EAE animals. We analyzed NF-kB pathway after the preventive (from 7^th^ and 21^th^ d.p.i.) and therapeutic (from 23^th^ and 36^th^ d.p.i.) treatment with IP7e.

The analysis of preventive administration disclosed a down-regulation of 16 target genes involved in the NF-kB signaling pathway in the spinal cord (see Table 1). These genes are responsible for cytokines and chemokines production, inflammation, immune response, apoptosis, type I interferon-responsive genes, development and differentiation of the nervous system and of lymphoid organs. Conversely the therapeutic treatment, starting from the 21^th^ d.p.i. is not able to induce alterations of the NF-kB downstream genes expression. These results indicate that only the preventive IP7e treatment, started before the disease onset, exerts an inhibitory effect on NF-kB signaling.

**Table 1:** List of spinal cord down-regulated genes after IP7e preventive administration in MOG_35–55_ induced EAE mice compared to vehicle-control.

Gene Name	Gene Symbol	Fold Regulation	p-value	Function
Complement component 3	C3	−6,29	0,036	Inflammation
Chemokine (C-C motif) ligand 12	Ccl12	−3,96	0,012	Cytokines and Chemokines
Chemokine (C-C motif) ligand 22	Ccl22	−2,80	0,034	Cytokines and Chemokines
Chemokine (C-C motif) receptor 5	Ccr5	−4,60	0,019	Inflammation
CD74 antigen	Cd74	−6,81	0,033	Apoptosis
Chemokine (C-X-C motif) ligand 1	Cxcl1	−2,30	0,016	Cytokines and Chemokines, development and differentiation of nervous system
Interleukin 1 alpha	Il1a	−5,24	0,033	Cytokines and Chemokines
Interleukin 2 receptor, alphachain	Il2ra	−3,31	0,013	Apoptosis, development and differentiation of limphoid
Lymphotoxin B	Ltb	−4,09	0,034	Cytokines and Chemokines, Immune Response
Reticuloendotheliosis oncogene	Rel	−2,62	0,018	TranscriptionFactor
V-relreticuloendotheliosis viral oncogene homolog A (avian)	Rela	−2,30	0,025	Transcription Factor (NF-kB signaling)
Selectin, endothelialcell	Sele	−4,14	0,002	NF-kB pathway
Selectin, platelet	Selp	−3,21	0,004	Inflammation
Signal transducer and activator of transcription 1	Stat1	−4,31	0,042	Transcription Factor, NF-kB pathway, Type I Interferon-Responsive genes
Tumor necrosis factor (ligand) superfamily, member 10	Tnfsf10	−2,35	0,024	Cytokines and Chemokines, Apoptosis, Immune Response, Type I Interferon-Responsive genes, NF-kB pathway
Vascular cell adhesion molecule 1	Vcam1	−2,12	0,046	Differentiation and differentiation of limphoid

Among the 84 analyzed genes of the NF-kB pathway, we evaluated the Nurr1 gene expression and did not observe any differences after the treatment. This concurs in with the mechanism of action of this compound that induces a Nurr1 signaling pathway activation without increased Nurr1 RNA transcription [28].

## Discussion

The present study is the first one focused on the effect of IP7e, a highly potent blood brain barrier crossing activator of the Nurr1 signaling pathway [28] on EAE in mice. The chosen EAE model allows an evaluation of the involvement of Nurr1 signaling in both acute and chronic stage. The course of the pathology in the model is characterized by an initial peak of neuronal and inflammatory signs followed by a chronic phase [29].

Since we previously reported a Nurr1 gene expression down-regulation in PBMCs and in particular in CD4+ T cells and monocytes obtained from MS patients compared to healthy controls (HC) [16]–[18], we decided to activate the Nurr1 signaling pathway in a murine model of MS to better characterize the role of this transcription factor on the EAE pathogenesis.

We found that preventive treatment with IP7e reduces the incidence and the severity of EAE. This novel compound is also able to attenuate inflammation and neurodegeneration in the spinal cord of EAE-treated mice. In our investigation, the activation of the Nurr1 signaling pathway significantly improved the outcome of EAE and suppressed the accumulation of immune cells i.e. T lymphocytes and macrophages in the spinal cord of EAE mice. It is known that autoimmune T cells and macrophages are essential for the initiation of EAE, in fact following immunization, encephalitogenic CD4+ T cells invade the parenchyma of the CNS to orchestrate the invasion of non-antigen-specific lymphocytes and macrophages and local inflammation process [30], [31]. This could explain why the therapeutic treatment with IP7e had no effect on EAE mice when the pathology had already occurred.

Our findings seem to contradict previous work, in which knocking down Nurr1 reverses autoimmune responses and ameliorates clinical symptoms in EAE [25]. These differences may be due to the dissimilar experimental procedure used to block or activate in Nurr1. In fact, Raveney and coworkers used small interfering RNA to inhibit Nurr1 up-regulation on the day of EAE induction, while we continuously treated EAE mice with an activator of the Nurr1 signaling pathway, starting when phenotypic EAE signs were not yet evident but the immunization process had already occurred.

A potential mechanism at the basis of the improvement of EAE observed after the preventive IP7e treatment could be represented by the block in the early phase of the disease of the pro-inflammatory NF-kB pathway by Nurr1 signalling activation. In fact, along with EAE improvement we observed down-regulation of NF-kB downstream genes after the preventive treatment. On the other hand, we did not report any effect on EAE condition and NF-kB pathway gene expression when we treated animals after the disease onset (i.e. therapeutic administration).

Our data and the evidences concerning the role of Nurr1 in T cells development and in its anti-inflammatory functions suggest a possible role of this activation pathway in the early phase of EAE, controlling the inflammation and the invasion of immunity components into the parenchyma. Therefore, the clinical implication on human MS could be the possibility to reduce the strength of single attack decreasing inflammation with IP7e, but further and accurate experiments are necessary to clarify this point.
